# Investigating the Effects of Acid Hydrolysis on Physicochemical Properties of Quinoa and Faba Bean Starches as Compared to Cassava Starch

**DOI:** 10.3390/foods13233885

**Published:** 2024-12-01

**Authors:** Anthony Halim, Peter J. Torley, Asgar Farahnaky, Mahsa Majzoobi

**Affiliations:** Discipline of Biosciences and Food Technology, RMIT University, Bundoora West Campus, Plenty Road, Melbourne, VIC 3083, Australia; s3909645@student.rmit.edu.au (A.H.); asgar.farahnaky@rmit.edu.au (A.F.)

**Keywords:** acid thinning, acid hydrolysis, cassava starch, faba bean starch, quinoa starch, emerging starches, underutilised starches

## Abstract

In response to the growing demand for high-quality food ingredients, starches from underutilised sources like quinoa and faba bean are gaining attention due to their unique properties and high tolerance to adverse environmental conditions. Acid hydrolysis is a well-established chemical method for producing modified starch with improved solubility, lower gelatinisation temperature, and reduced pasting viscosity. However, various outcomes can be achieved depending on the type of starch and modification conditions. This study comparatively investigated the effects of acid hydrolysis on the functional and physicochemical properties of emerging starches from quinoa and faba bean, with cassava starch serving as a reference from a leading source. The results demonstrated increased dietary fibre content across all three starches, with faba bean starch showing the most significant rise. Acid treatment also enhanced the crystallinity of the starches, with faba bean starch exhibiting the highest increase in relative crystallinity, which led to a shift towards higher temperatures in their thermal properties. Additionally, water solubility and oil adsorption capacity increased, while swelling power decreased following acid treatment. The acid treatment reduced the pasting properties of all samples, indicating that the modified starches were more resistant to heating and shearing in the rapid visco analyser. While quinoa starch gel remained soft after acid hydrolysis, the gel strength of cassava and faba bean starches improved significantly, making them suitable as plant-based gelling agents.

## 1. Introduction

In the modern food industry, there is an increasing demand for high-quality food ingredients to improve food product quality and safety. Starch, a ubiquitous carbohydrate polymer, plays an essential role as a food ingredient due to its wide affordability and versatility of functional properties [1]. Native starches are traditionally extracted from a few leading crops, such as corn, wheat, potato, rice, and cassava. However, food security and sustainability considerations drive further research to find alternatives, with amaranth, quinoa, and faba bean as emerging starch sources. Quinoa and faba bean starches were further researched in this paper as emerging starches, with benchmark to cassava starch as the leading source.

Cassava (*Manihot esculenta* Crantz), also known as tapioca, is a major crop in tropical and subtropical countries and a staple food. Cassava is highly resilient under harsh climatic conditions, is available all year round, and has simple extraction methods [2,3]. Cassava starch has been reported to have an amylose content ranging from 15.1 to 28.2% [4]. Its granules are typically oval or truncated at the ends, with the most common granule size reported being 7–20 μm [5,6]. Cassava starch has numerous advantages, such as being gluten-free and allergy-free; exhibiting higher water-swelling power and solubility than leading starches such as wheat, corn, and potato starches; and producing a clear gel, placing it as a valuable starch for modern food processing [7]. However, it produces a sticky and liquid-like gel upon cooling and is more susceptible to gelatinisation (low gelatinisation onset) and retrogradation, limiting its applications in some food products [8].

Quinoa (*Chenopodium quinoa* Willd.), a South American native crop, has emerged as a gluten-free and allergy-free superfood due to its excellent nutritional profile, containing high protein, essential amino acids, dietary fibre, and multivitamins [9]. Quinoa is a pseudo-cereal, with starch accounting for up to 70% of its dry weight. Quinoa starch has an amylose content ranging from 2.7 to 16.9% across different varieties and contains non-spherical or oblong-shaped granules that together form aggregates with a size range of 10–30 μm [10]. It has higher swelling power, peak viscosity, enzyme susceptibility, and lower retrogradation rate than other starches, including potato, wheat, and corn starches. However, it has a lower gelatinisation temperature and produces a soft, turbid gel during cooling [10]. These features may limit some of its food applications.

Faba bean (*Vicia faba* L.) is a winter legume crop with high nutritional value and is an emerging plant-based protein source [11]. With the increasing demand for plant-based protein from faba bean, the market for faba bean starch is also growing significantly. Faba bean starch has a high amylose content, ranging from 32.2 to 39.9% [12]. The granules are kidney or irregular, oval-shaped, with granules containing some crack features and a size of 11–48 μm [13]. Faba bean starch has good foaming capacity and stability, water absorption capacity, and higher resistant starch than other pulse starches. However, it has lower water solubility and swelling capacity than other pulse starches [14].

Various modification techniques are often employed, including physical, chemical, and enzymatic methods and their combinations, to improve the functional properties of starch and address some of its limitations [15]. Notably, the market value of modified starch significantly exceeds that of native starch [16], highlighting the growing demand for tailored functionality. Cassava and faba bean starch have been modified using physical modifications, including hydrothermal, irradiation, and ultrasound treatments. Their chemical modifications of faba bean starch using oxidation, cross-linking, substitution, and acid thinning have been reported [14,17]. Nonetheless, modification of quinoa starch has been less investigated. Some studies have indicated the success of octenyl succinic anhydride modification of quinoa starch as a stabilising agent for Pickering emulsions. Quinoa starch has also been physically modified by heat treatment to enhance emulsifying capacity and by high hydrostatic pressure to increase resistant starch content [10].

**Figure 1:**
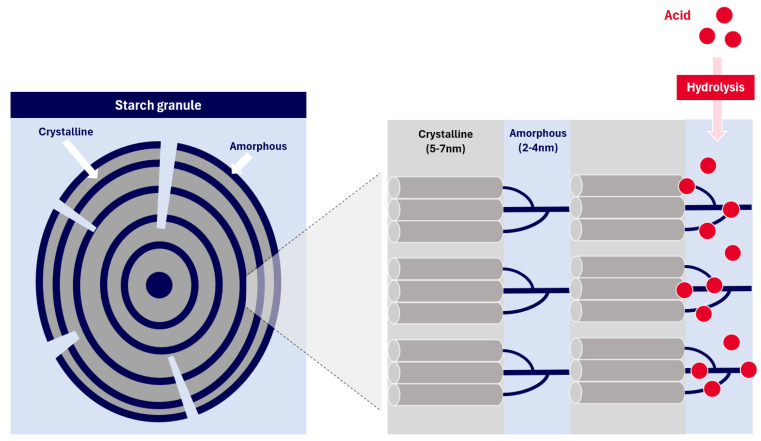
Visualisation of acid hydrolysis occurred in starch granules.

Acid hydrolysis is a well-established chemical approach for producing modified starch with improved solubility, lower gelatinisation temperature, and reduced pasting viscosity [18,19]. Under acidic conditions, Starch undergoes hydrolysis, involving the breakdown of glycosidic bonds. Hydrolysis (Figure 1) starts on the starch surface, with faster hydrolysis in the more loosely packed amorphous regions than the crystalline domains [20]. Many process parameters also influence the output of acid-treated starch, such as acid type, concentrations, time, and temperature used [21], which can be further customised to produce the desired properties of acid-treated starch. Diluted strong acids, like hydrochloric acid and sulfuric acid, are the most commonly used to produce hydrolysed starch from various botanical sources, such as corn, sorghum, potato, pea, and cassava. Acid-treated starch generally exhibits increased solubility, pasting clarity, crystallinity profile, decreased pasting properties, swelling power, textural properties, and gel stability [22,23,24]. Acid-treated starches are widely used in the confectionary industry as gelling agents. In recent years, research has extended to explore its application in the production of starch nanoparticle production [25,26,27], either on its own or in combination with other methods.

This study aims to extend the functional properties and applications of cassava, quinoa, and faba bean starches using acid hydrolysis. The physicochemical properties of these starches, including total dietary fibre, crystallinity, granule morphology, thermal profile, pasting properties, and textural profile analysis, were compared. This research will provide valuable insights into the added value of the emerging and underutilised starches for diverse applications in food, pharmaceutical, and other relevant industries.

## 2. Material and Methods

### 2.1. Materials

Native cassava starch (C-N) was obtained from Ingredion (Sydney, NSW, Australia). White quinoa flour was purchased from Bob’s Red Mill, Bio Living International Pty. Ltd., (Melbourne, VIC, Australia). Faba bean flour was a kind gift from Essantis, Smeaton, Victoria, Australia. The total Dietary Fiber kit (K-TDFR) and Glucose Content kit (K-GLUC) were purchased from Megazyme (Neogen Australasia, Ipswich, QLD, Australia). All chemical reagents used were purchased from Merck Sigma-Aldrich (Melbourne, VIC, Australia) with analytical grade, which included hydrochloric acid, natrium hydroxide, dimethyl sulfoxide, maleic acid, calcium chloride dihydrate, glacial acetic acid, and ethanol.

### 2.2. Starch Extraction from Faba Bean and Quinoa Flour

**Figure 2:**
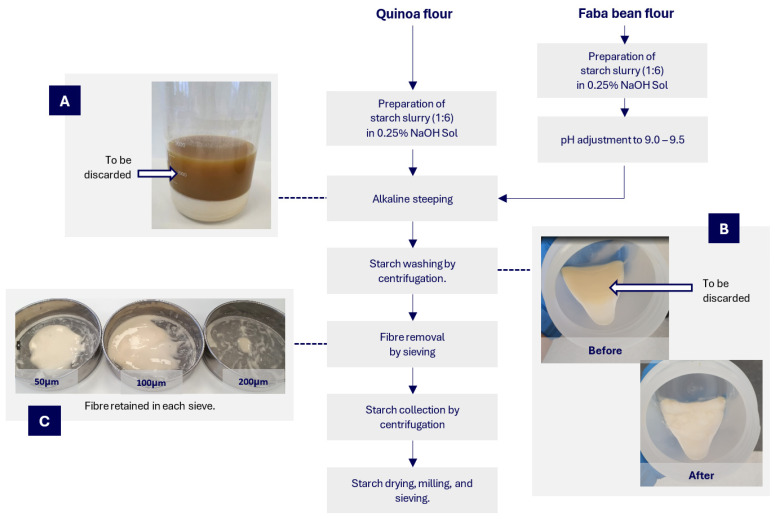
Process flowchart of alkaline steeping for extracting quinoa and faba bean flour: (**A**) the upper brownish layer was discarded after alkaline steeping; (**B**) the upper layer (slightly darker) was discarded after the washing and centrifugation step; (**C**) the cellulosic fibre fraction was removed by sieving.

Alkaline steeping was used to extract starch from quinoa flour [28], with modification, and faba bean flour [11], with modification (Figure 2). The process used for both starches was the same, except that pH adjustments were only performed in faba bean starch extraction.

The starch slurry was prepared by steeping 1 part of flour in 6 parts of 0.25% NaOH solution. During faba bean starch extraction, the pH was adjusted to 9.0 to 9.5 with 1 M NaOH. The slurry was then incubated (4 °C, 24 h). After incubation, the brownish upper layer was discarded (Figure 2B). The lower starch layer was resuspended in distilled water and sieved through sieves with sizes of 200, 100, and 50 μm. Any residues retained in the sieves were discarded. The sieved portions were reconstituted and mixed with approximately 50% distilled water and centrifuged (6000× *g*, 20 °C, 10 min; Sorvall LYNX 600, Thermo Fisher Scientific, Melbourne, VIC, Australia), and then the pale brown upper layer was manually removed (Figure 2C). The centrifugal washing process was repeated five times. The resultant was oven-dried at 45 °C for 24 h. After drying, the starch was ground using a 100 μm sieve (Ultra Centrifugal Mill ZM200, Retsch-Metrohm, Sydney, NSW, Australia) and further sieved through a sieve size of 100 μm. The starch extraction recovery rate was calculated at 35.7% for quinoa starch (Q-N) and 42.3% for faba beans (F-N).

### 2.3. Starch Modification

The acid hydrolysis process was performed using a method by [18], with modification. A starch slurry was prepared by mixing 150 g of starch in 250 mL of distilled water, and then 8.0 mL of 37% HCl was added to the starch slurry. The acidified starch slurry was incubated and shaken intermittently in a water bath (OLS26, Grant, Melbourne, VIC, Australia) at 50 °C for 6 h (namely AT6) or 24 h (namely AT24). At the end of incubation, the starch slurry was cooled in an ice bath to room temperature (25 °C), and then the pH was adjusted to 5.0 ± 0.2 using 1 M NaOH. The starch slurry was washed three times via centrifugation (6000× *g*, 10 min; Sorvall LYNX 600, Thermo Fisher Scientific, VIC, Australia) between each washing step. The acid-treated starch was dried in a fan-forced oven (45 °C, 12 h), milled using a 100 μm mesh size (Ultra Centrifugal Mill ZM200, Retsch-Metrohm, NSW, Australia), and further sieved using a 100 μm mesh size. Acid-treated starches were stored in air-tight glass containers for further analysis.

### 2.4. Total Dietary Fibre Analysis

Total dietary fibre was analysed according to the AOAC 991.43 procedure, with modification, using the Megazyme Total Dietary Fiber (K-TDFR) kit. The glucose content released due to the starch digestion using enzymes in the kit was determined using glucose oxidase–peroxidase (GOPOD), and reading was performed using UV-vis spectrometry (Lamda 35, PerkinElmer, Waltham, MA, USA) at 510 nm.

### 2.5. X-Ray Diffraction

The moisture content of all starch samples was equilibrated by incubating the starch powders over a 75% sodium chloride solution (3:1 in distilled water) inside the desiccator for three days at room temperature. The equilibrated samples were analysed using an X-ray diffractometer (D4 Endeavour, Bruker, Melbourne, VIC, Australia). Each sample was smoothly packed in a 10 mm round specimen holder. The analysis was performed with a generator set at 35 kV and 20 mA. The diffractograms were recorded on a continuously spinning sample of 2θ from 6.0° to 40.0°, with a scanning rate of 3.8°/min. The X-ray diffractograms were analysed using OriginPro 2021b (OriginLab Corporation, Northampton, MA, USA). All results were smoothed using the Savitzky–Golay algorithm provided in the OriginPro software, with parameters of polynomial order one and a window of 30. The baselines and peaks were integrated by pinning a connecting point at the base of the peak, using OriginPro’s spline algorithm to smooth the baseline. The relative crystallinity was calculated according to the following equation [29]:

(1) $$Relative\;crystallinity\;\left(\%\right)=\frac{Ac\;}{Aa+Ac}\times 100\%$$

where Ac is the crystalline region area, and Aa is the amorphous region area.

### 2.6. Thermal Properties

Differential scanning calorimetry (DSC) was used to determine the thermal behaviour of native and acid-modified starches based on the method by Pereira and Beleia [18]. The analysis was performed using DSC Q2000 (TA Instruments, Rydalmere, Australia). The dried samples were weighed into aluminium DSC pans and mixed with distilled water in a ratio of 1 to 3 (starch–water). The pans were hermetically sealed and stored at 4 °C for 24 h to allow the starch to hydrate. The prepared pans were scanned from 20 °C to 100 °C at 5 °C/min, with an empty pan as a reference.

### 2.7. Water Solubility and Swelling Power

Water solubility and swelling power were measured according to the method by Jiang et al. [30]. Each starch (0.1% *w*/*w*, dry basis) was suspended with distilled water in a centrifuge tube and then placed in a water bath (OLS26, Grant, VIC, Australia) at 85 °C for 30 min. While being heated, each tube vortex was mixed every 5 min. After heating, each tube was cooled to room temperature in an ice bath and centrifuged (3000× *g*, 15 min). The supernatant was carefully collected and dried (105 °C, 3 h). The weight of the precipitate and the dried supernatant was recorded. Water solubility and swelling power were calculated using the following equations:

(2) $$Water\;solubility\;\left(\%\right)=\frac{Ws\;}{Wo}\times 100\%$$

(3) $$Swelling\;power\;\left(g/g\right)=\frac{Wp-Wo\;}{Wo}\;$$

where (Wo) is the dry weight of starch, (Ws) is the weight of the dried supernatant, and (Wp) is the weight of the precipitate.

### 2.8. Oil Adsorption Capacity

The oil adsorption capacity was performed using a modified method by Wang et al. [31]. Starch (1.0 g, dry basis) was mixed with 10 mL of vegetable oil (Crisco, Sydney, NSW, Australia) and vortexed for 10 min. After standing for 10 min at room temperature, each sample was centrifuged (3000× *g*, 20 min). The separated oil was carefully discarded, and the tube was inverted on absorbent paper until there was no further oil drainage. The weight of the precipitate was then measured. The oil adsorption capacity was calculated as follows:

(4) $$Oil\;absorption\;capacity\;\left(\%\right)=\frac{\left(W_{p}-W_{o}\right)}{W_{o}}\times 100\%$$

where (W_o_) is the weight of dry starch, and (W_p_) is the weight of the precipitate.

### 2.9. Colour Analysis

The colour of starch powder samples was measured using a colourimeter (Minolta CR400, Minolta Corporation, Ramsey, NJ, USA). The colourimeter was calibrated before each use with a white reference plate. The measurements were performed in triplicate, and the CIE L*a*b* parameters were recorded. The relative difference between the two samples was calculated according to the following equation [29]:

(5) $$Relative\;color\;difference\;\left(\Delta E\right)=\sqrt{{\left(L_{1}^{*}-L_{2}^{*}\right)}^{2}+{\left(a_{1}^{*}-a_{2}^{*}\right)}^{2}+{\left(b_{1}^{*}-b_{2}^{*}\right)}^{2}}$$

where L* is lightness, a* is redness/greenness, and b* is yellowness/blueness.

### 2.10. Granule Morphology with Polarised Light Microscope

Polarised light microscopy was utilised to evaluate the morphology of granules based on method variation by Cai et al. [32]. A polarised microscope (Olympus BX53-P, Evident Scientific, Tokyo, Japan) was used with a 40× magnification. A polarised lens of 530 nm was used as the filter to capture the image.

### 2.11. Granule Morphology with Scanning Electron Microscopy (SEM)

The SEM was used as an additional method to analyse the granule morphology. All starch samples were coated with iridium (5 nm). The scanning electron microscopy (SEM) micrographs were captured with an FEI Quanta 200 Microscope (FEI, Salem, MA, USA). Cassava starch was observed at 20 kV, in a spot size 5.0, and a working distance of 9.4–10.8 mm. For quinoa starch, the parameters were 10 kV, a spot size of 4.0, and a working distance of 10.0 mm. Faba bean starch parameters were 30 kV, a spot size of 4.0, and a working distance of 10.8 mm.

### 2.12. Pasting Properties

Pasting properties were analysed using a rapid visco analyser (RVA 4500, Perten, Macquarie Park, Sydney, NSW, Australia) with the method developed by Kapusniak and Nebesny [33], with modifications. The starch slurries were prepared by mixing 3 g of starch in 25 g of distilled water (with a moisture content of 14%) using the Standard Method-1 profile. Peak, breakdown, setback, and final viscosity were determined from the RVA graphs, according to [34].

### 2.13. Texture Profile Analysis of Starch Gels

Gel texture profile analysis was performed using a texture analyser (TA.XT Plus Texture Analyser, Stable Micro Systems, Godalming, UK) according to the method by Pereira and Beleia [18], with some modifications. The starch gels acquired from the RVA tests were poured into a Plexiglass cylinder with a height of 11 mm and a diameter of 30 mm and stored in sealed containers for 24 h at 4 °C. The analysis was performed using a cylindrical probe (P/45, aluminium), with a test, pre-test, and post-test speed of 2 mm/s, 10 mm/s, and 10 mm/s, respectively. A double compression test was conducted with a compression ratio of 40% and trigger force of 5 g, with a resting time of 10 s between the two compressions.

### 2.14. Statistical Analysis

All numerical results are averages of at least triplicates, except for DSC and XRD, which were performed in duplicates. The results are presented as the mean ± standard deviation. Graphics were visualised using Microsoft^®^ Excel^®^ for Microsoft 365 MSO (Version 2308 Build 16.0.16731.20542) 64-bit and OriginPro 2021b (OriginLab Corporation, Northampton, MA, USA). One-way analysis of variance (ANOVA) with Tukey’s test (*p* ≤ 0.05) was used in the result analysis using Minitab software (version 18.1, Minitab Inc., Pennsylvania, US).

## 3. Results and Discussion

### 3.1. Total Dietary Fibre (TDF)–Total Indigestible Carbohydrate (TIC)

The TDF content of the samples indicated the starch portion that remained undigested by the action of the starch digestive enzymes and behaved as a dietary fibre. As shown in the TDF results in Table 1, F-N was identified as the highest TDF content among native starches, at 5.48%, followed by C-N and Q-N, at 5.21% and 5.23%, respectively. Meanwhile, the acid-treated results showed a slight increase compared to the native results, which was statistically significant. Faba bean starch treated for 24 h recorded the highest value of TDF (6.03%), while cassava starch treated for 24 h showed the lowest TDF content (5.53%). These findings align with previous research, which reported similar observations of an increase in the indigestible carbohydrate fraction following the acid treatment of corn starch [35,36]. The differences between the TDF of starches from various sources can be attributed to their intrinsic difference in granular characteristics and porosity, amylose and amylopectin content, molecular structure and configuration, and degree of crystallinity. Understanding these parameters’ effects on the TDF needs further investigation.

**Figure 3:**
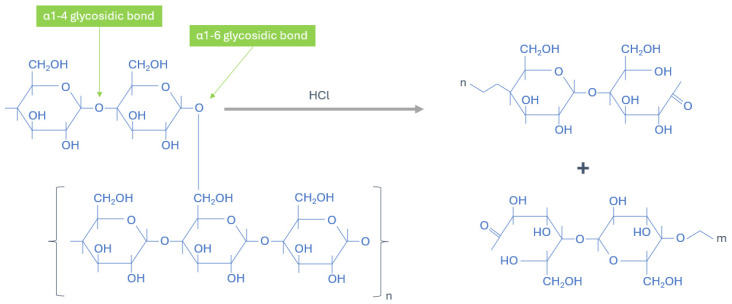
The chemical reaction of acid hydrolysis of starch molecules.

When acid is added to starch, it gradually penetrates into the starch granules and initiates the hydrolysis of starch molecules. Most starches demonstrate two stages of the hydrolysis reaction, which begin with rapid hydrolysis on the surface and amorphous region (mostly amylose) and subsequent hydrolysis at a slower rate on the crystalline area, mainly amylopectin (Figure 3). The amorphous areas contain more accessible binding sites, allowing for a faster hydrolysis rate. Meanwhile, the dense arrangement of the starch chain in the crystalline area is hypothesised to hinder the penetration of hydrogen ions and lead to a slower hydrolysis rate. As a complementary, the second hypothesis proposes that converting glucopyranose rings from the chair conformation to the half-chair conformation is essential for the cleavage of glycosidic bonds and is attributed to the slower pace of hydrolysis [20].

Depending on the extent of hydrolysis, the crystalline area exhibits an increase at the early phase of treatment with mild acid, which is attributed to the reduction in the amorphous region. This effect increases the TDF content due to more crystalline area, which can withstand digestion by gut enzymes [37]. Further research can be performed to evaluate the amylose–amylopectin ratio and the structural differences in these starches to elaborate a more comprehensive discussion of results.

### 3.2. X-Ray Diffraction (XRD) and Relative Crystallinity (RC)

**Figure 4:**
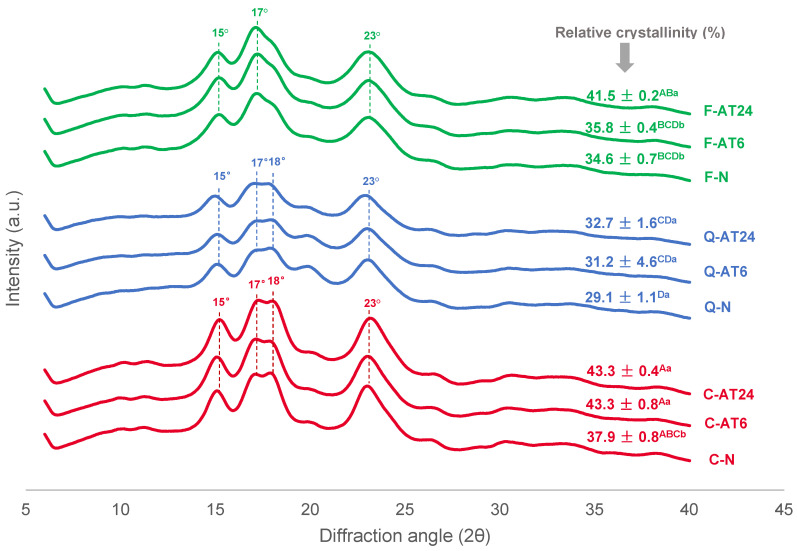
XRD profiles of native and acid-treated cassava, quinoa, and faba bean starch at 6 h and 24 h treatment time. The % relative crystallinities are presented in mean ± standard deviation. Values are mean ± standard deviation. Different superscript uppercase letters indicate significant differences (*p* < 0.05) between values in one column. Different superscript lowercase letters indicate significant differences (*p* < 0.05) between the same botanical source.

Starch molecules exhibit a semi-crystalline structure, with the branched amylopectin chains arranged into crystalline lamellae, forming double helices. These crystalline double helices alternate with amorphous regions predominantly consisting of branch points. A crystalline region and an adjacent amorphous region constitute a “cluster” in amylopectin, measuring approximately 9 nm in length [14]. The XRD result can provide a crystallinity profile for native and acid-treated starches. The XRD diffractograms of all samples are presented in Figure 4. Both cassava and quinoa starches were observed with a distinct diffraction intensity at peaks of 15°, 17°, 18°, and 23°, which indicates an A-type crystal pattern characteristic. Previous studies have also reported A-type crystallinity in cassava starch [18,38] and quinoa starch [39,40]. Meanwhile, faba bean starch exhibited a broader diffraction peak at 15°, 17°, and 23°, indicative of a mixed A- and B-type crystal structure. This C-type crystallinity is characteristic of legume starches, aligning with the findings of Guo et al. [41] and He and Wei [42]. Starch crystallinity is attributed to the portion of crystalline lamellae formed by the amylopectin double helical structure of each botanical source studied. The RC of tested starches were in the order of cassava starch (37.9%) > faba bean starch (34.6%) > quinoa starch (29.1%). The differences between the RC values of different starches are related to their differences in the granular size and composition, amylopectin content, the ratio of short- to long-chain amylopectin molecules, and amylose–lipid complex [43]. The low RC of quinoa starch may indicate the weak stability of the crystalline cluster due to the significant number of short chains that hinder the formation of the stable crystalline network [44]. The peak at approximately 0.44 nm (d-spacing) in the XRD spectrum is characteristic of amylose–lipid inclusion complexes. This peak is not prominent for quinoa starch, suggesting a low level of amylose–lipid complexation [10].

Acid treatment did not change the crystalline pattern of the starches; however, it led to an increase in RC of the samples, with faba bean starch showing the highest increase among the studied starches. Studies on other starches, such as achira starch, reveal a decrease in crystallinity during acid hydrolysis, followed by a partial recovery over extended hydrolysis periods. This study suggests that the impact of acid hydrolysis on crystallinity can vary depending on the starch source and the duration of hydrolysis [45]. Several hypotheses have been proposed to explain the increased crystallinity observed during the early stages of acid hydrolysis. Firstly, the cleavage of amylose chains traversing amorphous regions may enable the reorganisation of the newly released chain ends into more ordered crystalline structures. Secondly, restructuring the crystalline regions during acid hydrolysis could enhance crystallinity by partially filling the water channels within crystallite cavities with double helices. Lastly, the increased crystallinity may also arise from the retrogradation of hydrolysed free amylose, which forms double helices that subsequently arrange into acid-resistant crystalline regions [17]. It was found that the acid treatment did not statistically impact the RC value of quinoa starch, which indicates that acid treatment less affected the amylopectin crystalline regains of the quinoa starch. The effect can be caused by the compact packing of starch molecules within the starch granules, which prevents acid penetration and further acid hydrolysis resistance of quinoa starch granules. Further studies are required to confirm this hypothesis.

### 3.3. Thermal Properties

**Figure 5:**
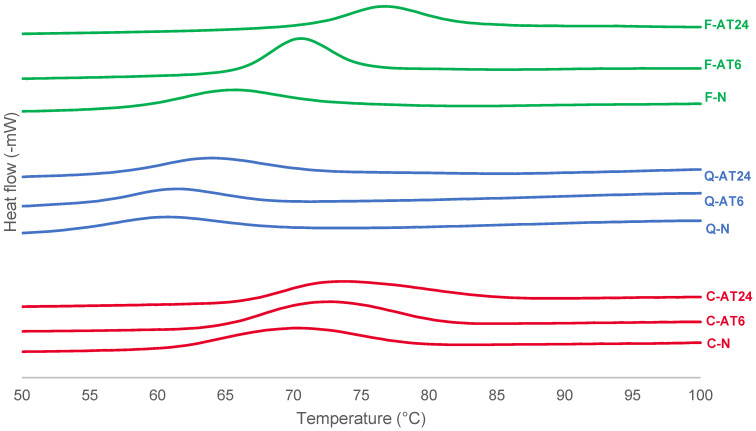
DSC profiles of native and acid-treated cassava, quinoa, and faba bean starches at 6 h and 24 h processing time.

The analysis of thermal properties with DSC can provide insight into starch’s thermal behaviour during gelatinisation. The thermal properties’ data are presented in Table 2, with details of onset temperature (To), peak temperature (Tp), conclusion temperature (Tc), and enthalpy change (ΔH), with the DSC profiles visualised in Figure 5. Overall, the acid treatment applied in this research caused a shift towards higher temperatures for To, Tp, and Tc. Although the ΔH values tended to increase for cassava and quinoa starches, the results did not reveal statistically significant (*p* < 0.05) differences between the native and acid-treated starches within each starch type and processing time. Quinoa starch had the lowest gelatinisation temperature and enthalpy, indicating that this starch is more easily gelatinised than cassava and faba bean starches. This low gelatinisation temperature range can have processing implications for quinoa starch as an emerging starch source. Correlation analysis shows that the gelatinisation temperatures and ΔH are closely associated with the fine structure of amylopectin [46]. For instance, the high proportion of short unit chains, particularly the Afp chains, combined with the low proportion of long unit chains in quinoa amylopectin, may account for the low gelatinisation temperatures of quinoa starch. Additionally, quinoa starch has no or very low amylose–lipid complex, leading to a less crystalline structure and lower gelatinisation temperature [10].

The results were aligned with XRD, which suggested that the acid-treated starches had improved the crystallinity levels for cassava and faba bean starches but were less effective for quinoa starch.

### 3.4. Granule Morphology

**Figure 6:**
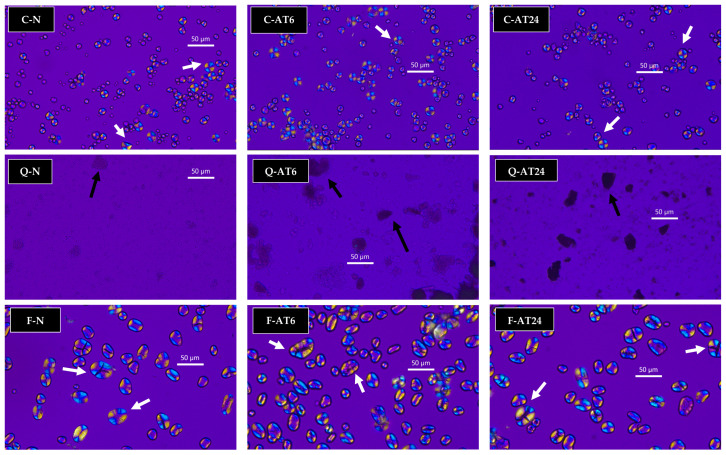
Polarised light microscopy images of cassava (C), quinoa (Q), and faba bean (F) starches. Legend: native starch, N; acid-treated for 6 h, AT6; acid-treated for 24 h, AT24.

**Figure 7:**
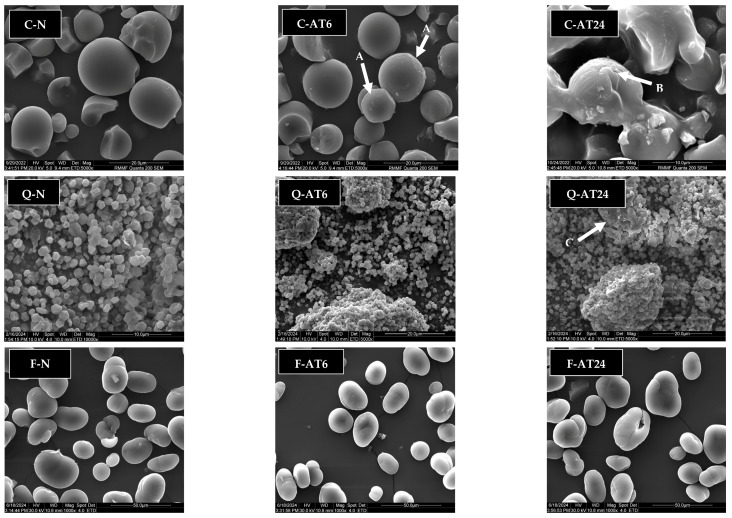
SEM results for images of cassava (C), quinoa (Q), and faba bean (F) starches. Legend: native starch, N; acid-treated for 6 h, AT6; acid-treated for 24 h, AT24.

Two characterisation methods were utilised to study granule morphology, namely polarised light microscopy, with the results shown in Figure 6, and scanning electron microscopy (SEM), with its results shown in Figure 7.

### 3.5. Colour Analysis

From overall polarised light microscopy results, it can be inferred that faba bean starch had the visually largest granule size, followed by cassava and quinoa starches. Cassava and faba bean starches exhibited clear observable birefringence of a Maltese cross (Figure 6, shown in white arrow) but no apparent observable polarised properties for quinoa starch, only clustered granules (Figure 6, shown in black arrow). The polarised results were highly correlated to the aforementioned crystallinity profiles, where cassava and faba bean starches had higher crystallinity than the quinoa starch. Additionally, quinoa starch granules were too small to be adequately observed under the polarised light microscope; thus, SEM is more efficient in studying the morphological changes in quinoa starch.

According to SEM images, cassava starch granules varied in size and shape from irregular to oval granules with truncated ends and smooth surfaces. Roughening surfaces (Figure 7 C-AT6 in arrow A) in 6 h treatment and granule deformation (Figure 7 C-AT24 in arrow B) in 24 h treatment were observed in cassava starch. The findings align with [18], who reported that acid hydrolysis led to the roughening of the cassava starch surface.

Small granules with irregular to oblong shapes in agglomerated form were observed in quinoa starch, which aligns with the findings reviewed previously, indicating that quinoa starch has aggregated shapes, with each aggregate containing 14,000 to 20,000 individual starch granules [10,43,47]. It has been indicated that the agglomerated structure of quinoa starch is maintained by a protein network [43].

Acid treatment resulted in more deformed clustered granules in 24 h treatment (Figure 7 Q-AT24 in arrow C). Velásquez-Castillo et al. [43] also reported a similar deformation for quinoa starch after extensive hydrolysis to produce starch nanocrystals, with porous matter on the surface due to extensive acid hydrolysis.

**Figure 8:**
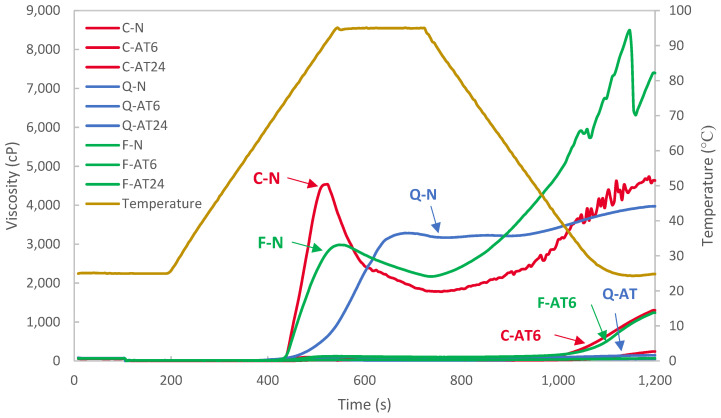
RVA graphs for native and acid-treated cassava, quinoa, and faba bean starches at 6 h and 24 h processing time.

Meanwhile, faba bean starch displayed beany to oval shapes with cracked surfaces of granules. The observations are consistent with previous reports [12,13]. The acid-treated faba bean starch exhibited slight changes in the granule morphology. Figure 8 shows some minor cracks on the faba bean starch granules, while most of the granules appear intact. Previous studies have shown that the hydrolysed faba bean starch’s granular morphology was not significantly disrupted, but some agglomerations were observed [14]. The implication of these findings for faba bean starch was significant, given that the acid treatment can significantly modify crystallinity and thermal profile without disrupting its granular morphology.

Table 3 outlines the colour analysis results for native and acid-treated cassava, quinoa, and faba bean starches. The L* (lightness), a* (red–green), and b* (blue–yellow) values obtained using the CIE Lab* system are reported. The impact of acid hydrolysis on the colour profile of the starches was evaluated.

Acid treatment significantly altered the colour parameters. Cassava starch was less yellow (lower b* value) and exhibited whiter powder for acid-treated results than the native. Meanwhile, the acid-treated quinoas were observed to be significantly less white, with a more yellow colour change. Like cassava, faba bean was observed to have a whiter appearance with a colour change towards a less yellowish colour.

Depending on the application, acid treatment can cause a shift towards a whiter colour for the cassava and faba bean starch, making it more desirable for food applications. However, it was less desirable for quinoa as the acid treatment made it yellowish and darker in appearance. The observed differences related to the impact of acid hydrolysis on the colour of the starches may be attributed to variations in their inherent properties, particularly particle size and morphology. Quinoa starch typically has smaller granules, with a range of 1–3 μm [10], compared to cassava and faba bean starches. Smaller particle size translates to a larger surface area-to-volume ratio. This increased exposure could lead to a greater hydrolysis rate to glycosidic linkage by acid and significantly affect the extent of browning. A similar finding has been reported for sorghum starch [48]. For cassava and faba bean starch, the larger particle size might hinder acid penetration into the granules, leading to a milder effect on the colour profile than quinoa starch.

### 3.6. Water Solubility (WS), Swelling Power (SP), and Oil Adsorption Capacity (OAC)

Table 4 compiles the results of water solubility, swelling power, and oil adsorption capacity of native and acid-treated cassava, quinoa, and faba bean starches at 6 h and 24 h processing times. Generally, the effect of acid treatment increased water solubility and oil adsorption capacity but decreased the swelling power. The highest solubility among native starches was exhibited by quinoa, with 10.4%, followed by cassava and faba bean starches, at 6.8% and 5.3%, respectively. In contrast, the swelling power of quinoa tended to be lower, with a range of 0.4–3.3%, compared to other starches.

Many factors contribute to solubility and swelling capability, such as granule size, the chain-length distribution of starch molecules, chemical composition, and amylose–amylopectin ratio. The presence of fibre and protein in starches, particularly quinoa starch, along with the hydrophobic nature of these molecules, may contribute to increased water solubility. The intact structure of amylopectin is considered crucial for starch granule swelling and water-holding capacity. When the amylopectin structure is disrupted, the formation of an intact network becomes impossible, and the damaged chains are more likely to dissolve, as they can no longer trap water or oil. Additionally, acid-hydrolysed starch granules are believed to become fragile and fragment upon heating in water rather than swelling [20].

When starches are treated with acid, the hydrolysis kinetics begins from the surface and amorphous region of starches. Acid treatment impairs both the hydrophilic and hydrophobic capacities of native starch. This process reduces water absorption capacity due to an increase in the crystalline region and a decrease in the amorphous region of the starch granules. The reduction in the number of available binding sites lowers the water-binding capacity. Acid treatment can also fragment starch molecules, particularly amylose, in the amorphous regions of the granules. This process leads to the formation of smaller molecules with increased water solubility but reduced water-holding capacity. Several studies [17,18,20,49] reported the same results for cassava starch and faba bean starch, but limited research is available on quinoa starch in mildly acidic treatment. The results indicated that hydrolysis was more significant in quinoa starch, with a significant increase in solubility and reduced water-swelling capability. This can be related to the small granule size and amylose content, lower molecular packing density, and the high ratio of short-to-long amylopectin chains in quinoa starch [43]. Meanwhile, the faba bean starch also showed a significant decrease in swelling power.

A trend of increase was observed for the OAC results of acid-treated starch, but it was not statistically significant for cassava and quinoa starches. Acid treatment can create more internal granular space by reducing the amorphous regions to entrap more oil physically. Meanwhile, the effects of acid treatment on faba bean starch were observed to be statistically significant regarding OAC improvement. Faba bean starch has been reported to have a C-type crystal, as well as a loosely B-type and a more compact A-type combination. In acid treatment, the B-type is the preferred type for hydrolysis, as it allows more oil to interact with the structure, leading to more oil absorption [50]. In addition to starch as the primary component, the tested samples contained approximately 5% dietary fibre and a trace amount of protein (1.5–2%). Acid hydrolysis may influence the molecular structure and water affinity of these components and their capacity to interact with water and oil, which warrants further investigation.

### 3.7. Pasting Properties

The amylograph profiles are overlayed in Figure 8, and the results are detailed in Table 5. Among the native starches, the highest peak viscosity was observed in cassava, with 4475 cP, followed by quinoa with 3242 cP and faba bean with 2956 cP, and the highest final viscosity was measured for faba bean starch, with 8344 cP. The lowest breakdown viscosity of quinoa starch indicates its high resistance to continuous shearing and heating, followed by faba bean and cassava starches. Quinoa starch also showed the lowest final viscosity and lower tendency towards retrogradation. A decreasing trend of pasting viscosity was observed in all starches treated with acid thinning, with the lowest value observed in Q-AT24. Similar results have been reported for other acid-treated starches from sweet potato, lotus stem, and green banana starches [49].

The pasting viscosity explains the capability of starch granules to uptake the water and swell. A lower peak viscosity indicates a lower degree of swelling of starch during gelatinisation. As reported by Ulbrich et al. [19] for corn starch, the acid-thinning was capable of debranched amylopectin of the starch structure due to the tendency of cleavage of an α-1,4-glycosidic bond to react with the acid. Furthermore, the structural network of amylopectin is disrupted by acid hydrolysis to retain water and swell [51]. This finding is also in agreement with the decreasing swelling capacity (see Table 4) and consistent with a previous report on acid-treated cassava [18], indicating reduced pasting properties. However, limited research has been reported for the pasting properties of acid-treated quinoa and faba bean starches, with only a similar amylograph profile to those reported in [23] and [12] for native faba bean starch, according to which the final viscosity was significantly higher than the gelation peak viscosity. All the acid-treated starches showed higher final viscosities than their peak viscosities. The results indicate the degradation of starch molecules by acid treatment and the formation of smaller molecules, which can readily form a network and trap water to form a gel structure upon cooling stage during the RVA test. Similar results have been reported for green banana, lotus stem, and sweet potato starches [49]. Overall, acid treatment could significantly change the pasting properties of the tested starches. The treatment improved starch tolerance to high shear and temperature and reduced its retrogradation.

Further studies exploring the relationship between the degree of hydrolysis, the fibre content of the tested starches, and the resulting changes in pasting properties could yield valuable insights. Additionally, studies correlating pasting properties with freeze–thaw stability could provide a more holistic understanding of how acid hydrolysis influences starch functionality in frozen food applications.

### 3.8. Textural Property Analysis (TPA) of Starch Gel

**Figure 9:**
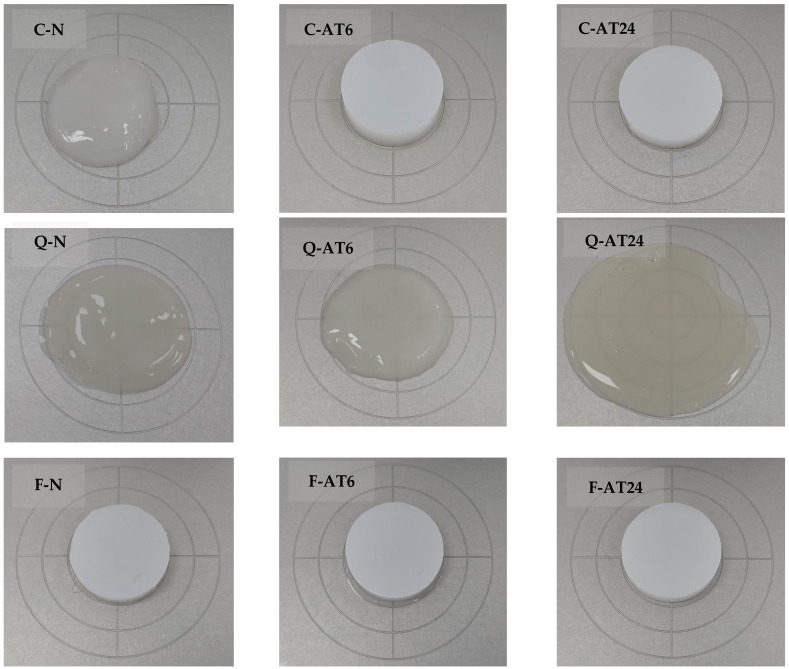
Gel results in texture profile analysis for cassava [native (C-N), acid-treated 6 h (C-AT6), and acid-treated 24 h (C-AT24)], quinoa [native (Q-N), acid-treated 6 h (Q-AT6), and acid-treated 24 h (Q-AT24)], and faba bean [native (F-N), acid-treated 6 h (F-AT6), and acid-treated 24 h (F-AT24)].

As shown in Figure 9, native cassava produced a sticky and soft gel that could not hold its structure. The results indicated the poor gelling properties of cassava starch as a leading starch, which can limit its food applications as a potent thickening agent. However, after acid treatment, its gelling properties significantly improved, and a strong gel was formed, indicating the acid treatment’s success in enhancing cassava starch’s gelling properties. This finding is based on the high industry application of cassava starch. Acid treatment could significantly improve cassava starch’s poor gel properties, resulting in a firm and self-sustaining gel. Measurement results of the gel firmness of the acid-treated cassava starch (Table 6) showed that the firmness for C-AT6 and C-AT24 was not significantly different. Thus, to achieve a firm gel, the 6 h acid treatment was sufficient to enhance the gelling properties of cassava starch.

Native faba bean starch could form a strong firm gel with a firmness of 1536.7 g, with no significant difference from the results of 1255.6 g of F-AT6. However, there was a significant reduction of firmness to 316.8 g for F-AT24, indicating the negative impact of long acid treatment on faba bean starch gel formation. The cohesiveness of cassava starch was not significantly affected by the acid treatment applied for 6 h and 24 h. Meanwhile, a decreasing trend of gel cohesiveness was observed in faba bean starch, with a significant change observed starting at 24 h treatment, indicating the reduced capability of faba bean starch gel to maintain its structure over a longer treatment time. This feature highlights the potential of native and acid-treated faba bean starch as underutilised natural gelling agents for various food products. Native quinoa starch could not form a gel and remained the same after acid treatment. This result indicates quinoa starch’s resistance to acid treatment and therefore holds promise in further applications in acidic foods where strong gel is not required, such as smooth and soft gel plant-based foods.

The effect of acid hydrolysis on the gelling properties of starch depends on the severity of the process and the chemical composition of starch. Mild acid hydrolysis (applied in this research) primarily involves debranching, opening up the complex structure in the amorphous region, and the limited degradation of large molecules (still maintaining a high degree of polymerisation). This process results in the formation of more linear molecules. Under these conditions, starch molecules can better form inter- and intra-interactions with other starch molecules and water, forming a three-dimensional network that leads to a firmer gel. Harsh hydrolysis conditions often lead to starch dextrinisation, the destruction of starch crystalline structure, and the formation of small molecules (dextrins), which are more water-soluble and unable to form a gel. The amylose-to-amylopectin ratio is also a key factor influencing starch gel texture [24]. Acid thinning debranches amylopectin, effectively increasing the amylose-to-amylopectin ratio. This shift results in a more rigid gel structure for cassava and faba bean starches. This finding aligns with the increased relative crystallinity observed in these starches after acid treatment. However, quinoa starch also exhibited increased relative crystallinity despite its low amylose content, suggesting that other factors, such as molecular characteristics and granular size, may contribute to the observed changes. Further investigation is needed to explain these factors.

Compared to other starches, quinoa starch contains much lower amylose content, with most references reporting a range of 2.7–16.9%, and amylopectin has a higher number of short single chains and a very low number of long single chains [10]. Due to the small size of its starch molecules, native quinoa starch may struggle to form a firm gel. Acid treatment could further reduce the molecular size of quinoa starch, resulting in an additional decline in its gelling properties. Additionally, the small individual particle size of quinoa starch (1–3 μm) may limit its ability to entrap water and swell, unlike cassava and faba bean starch.

The tested starches contained approximately 5% dietary fibre, so some of the observed changes may be attributed to the effects of acid hydrolysis on the molecular structure of the fibre and its subsequent interactions with water and starch molecules. This aspect warrants further investigation.

## 4. Conclusions

Although native quinoa and faba bean starches are underutilised, they have some unique features that can place them in a special place among competitors. These features can be further improved through acid treatment. The main changes observed by acid treatment of these starches included increased total dietary fibre content, increased resistance to continuous shearing and heating, decreased retrogradation, and increased starch solubility and oil-holding capacity. Notably, significant changes in gelling properties were observed, particularly in cassava starch, which could not form a gel in its native form but produced a strong gel after acid treatment. Faba bean starch, in native and acid-thinned forms, demonstrated strong gel formation, making it a promising novel gelling agent for food products, especially plant-based products. In contrast, quinoa starch exhibited more resistance to the acid treatment applied in this research, maintaining its weak gelling properties, which could be advantageous in products where soft gels are desirable, such as desserts and plant-based dairy and meat alternatives.

Future research should focus on optimising acid modification conditions to selectively hydrolyse the amorphous regions of starch while minimising the impact on the crystalline areas. Achieving this balance could extend the application of these starches as fibre fortifiers in food, enhancing nutritional content without significantly altering food viscosity or texture.

## Figures and Tables

**Table 1:** Total dietary fibre (TDF) levels for cassava, quinoa, and faba bean for native or acid-treated (6 h or 24 h) starches.

Starch		TDF (%)
Cassava	Native	5.209 ± 0.024 ^Ec^
	6 h	5.792 ± 0.003 ^Ca^
	24 h	5.531 ± 0.006 ^Db^
Quinoa	Native	5.230 ± 0.003 ^Ec^
	6 h	5.813 ± 0.052 ^Cb^
	24 h	5.916 ± 0.003 ^Ba^
Faba bean	Native	5.479 ± 0.006 ^Dc^
	6 h	5.895 ± 0.003 ^Bb^
	24 h	6.027 ± 0.001 ^Aa^

Values are mean ± standard deviation. Different superscript uppercase letters indicate significant differences (*p* < 0.05) between values in one column. Different superscript lowercase letters indicate significant differences (*p* < 0.05) between the same botanical source.

**Table 2:** Thermal properties of native and acid-treated (6 h or 24 h) cassava, quinoa, and faba bean starches.

Sample		To (°C)	Tp (°C)	Tc (°C)	ΔT (Tc-To) (°C)	ΔH (J/g)
Cassava	Native	60.7 ± 0.1 ^Cb^	69.6 ± 0.2 ^Da^	81.2 ± 1.1 ^BCa^	20.5 ± 0.1 ^ABCa^	12.9 ± 0.3 ^ABa^
	6 h	64.3 ± 0.5 ^Bab^	71.9 ± 0.1 ^BCb^	85.5 ± 3.8 ^ABa^	21.3 ± 0.3 ^ABa^	14.8 ± 1.4 ^Aa^
	24 h	65.2 ± 1.4 ^Ba^	73.7 ± 0.3 ^Bc^	86.9 ± 0.3 ^Aa^	21.7 ± 1.8 ^ABa^	14.2 ± 1.5 ^Aa^
Quinoa	Native	52.2 ± 0.8 ^Fb^	60.9 ± 1.2 ^Fa^	72.5 ± 0.5 ^Db^	20.3 ± 0.3 ^ABCab^	8.4 ± 0.4 ^Cb^
	6 h	54.5 ± 0.1 ^EFab^	61.3 ± 0.1 ^Fa^	72.3 ± 1.0 ^Db^	17.8 ± 1.0 ^ABCb^	8.0 ± 0.2 ^Cb^
	24 h	56.1 ± 0.5 ^DEa^	63.9 ± 0.6 ^Ea^	77.7 ± 0.3 ^CDa^	21.7 ± 0.2 ^ABa^	10.3 ± 0.5 ^BCa^
Faba bean	Native	57.6 ± 0.2 ^Dc^	65.2 ± 0.1 ^Ec^	80.1 ± 0.1 ^BCb^	22.5 ± 0.3 ^Aa^	10.9 ± 0.1 ^BCa^
	6 h	65.8 ± 0.1 ^Bb^	70.4 ± 0.1 ^CDb^	81.2 ± 0.4 ^BCb^	15.4 ± 0.3 ^Cb^	11.8 ± 1.1 ^ABa^
	24 h	70.5 ± 0.1 ^Aa^	76.7 ± 0.2 ^Aa^	87.5 ± 0.7 ^Aa^	17.0 ± 0.6 ^BCb^	10.5 ± 0.1 ^BCa^

Numerical values are presented in mean ± standard deviation. Different superscript uppercase letters indicate significant differences (*p* < 0.05) between values in one column. Different superscript lowercase letters indicate significant differences (*p* < 0.05) between the same botanical source. To is an onset temperature, Tp is the peak temperature, Tc is the conclusion temperature, AT is the gelatinisation temperature range, and ΔH is the gelation enthalpy.

**Table 3:** Colour analysis results (CIE L*a*b* system) for native or acid-treated (6 h and 24 h treatment) cassava, quinoa, and faba bean starches.

Samples		L*	a*	b*	ΔE
Cassava	Native	97.75 ± 0.01 ^Cb^	0.33 ± 0.01 ^Cc^	3.39 ± 0.01 ^Ca^	Reference point
	AT6	98.17 ± 0.09 ^Cb^	0.45 ± 0.01 ^Aa^	2.53 ± 0.03 ^Eb^	0.97 ± 0.01 ^BCa^
	AT24	97.93 ± 0.01 ^Ba^	0.38 ± 0.01 ^Bb^	2.50 ± 0.01 ^Eb^	0.44 ± 0.41 ^Ca^
Quinoa	Native	97.52 ± 0.01 ^Da^	0.01 ± 0.01 ^Eb^	2.80 ± 0.04 ^Dc^	Reference point
	AT6	96.97 ± 0.01 ^Eb^	0.01 ± 0.01 ^Eb^	3.99 ± 0.02 ^Bb^	1.31 ± 0.02 ^BCb^
	AT24	93.97 ± 0.11 ^Fc^	0.05 ± 0.01 ^Da^	7.26 ± 0.27 ^Aa^	5.70 ± 0.25 ^Aa^
Faba bean	Native	97.84 ± 0.02 ^BCc^	−0.57 ± 0.01 ^Gc^	3.73 ± 0.01 ^Ba^	Reference point
	AT6	98.21 ± 0.02 ^Aa^	−0.10 ± 0.01 ^Fa^	1.39 ± 0.01 ^Fb^	1.84 ± 0.50 ^Ba^
	AT24	98.11 ± 0.03 ^Ab^	−0.13 ± 0.01 ^Fb^	1.39 ± 0.00 ^Fb^	1.81 ± 0.50 ^Ba^

Values are mean ± standard deviation. Different superscript uppercase letters indicate significant differences (*p* < 0.05) between values in one column. Different superscript lowercase letters indicate significant differences (*p* < 0.05) between the same botanical source. (L*) is represents sample lightness, (a*) indicates a green-red value of the sample, and (b*) indicates a blue-yellow value of the sample. ΔE is the relative colour difference percentage.

**Table 4:** WS, SP, and OAC results for native and acid-treated cassava, quinoa, and faba bean starches at 6 h and 24 h processing time.

Sample		Water Solubility (%)	Swelling Power (g/g)	Oil Adsorption Capacity (%)
Cassava	Native	6.8 ± 0.3 ^DEb^	9.0 ± 0.8 ^Aa^	69.5 ± 1.6 ^BCb^
	6 h	9.1 ± 0.7 ^CDab^	5.6 ± 0.4 ^BCb^	72.9 ± 3.6 ^BCab^
	24 h	10.7 ± 1.6 ^Ca^	3.8 ± 0.7 ^CDc^	76.6 ± 1.7 ^Ba^
Quinoa	Native	10.4 ± 0.9 ^Cb^	3.3 ± 0.2 ^DEa^	67.2 ± 2.9 ^Ca^
	6 h	14.4 ± 2.5 ^Bb^	1.8 ± 0.4 ^DEFb^	70.5 ± 2.0 ^BCa^
	24 h	18.7 ± 0.7 ^Aa^	0.4 ± 0.1 ^Fc^	71.4 ± 1.4 ^BCa^
Faba bean	Native	5.3 ± 0.5 ^Eb^	7.4 ± 2.0 ^ABa^	58.9 ± 2.8 ^Dc^
	6 h	7.7 ± 0.6 ^CDEa^	2.3 ± 0.4 ^DEFb^	69.2 ± 3.9 ^BCb^
	24 h	8.9 ± 0.3 ^CDa^	1.1 ± 0.4 ^EFb^	91.5 ± 3.1 ^Aa^

Numerical values are presented in mean ± standard deviation. Different superscript uppercase letters indicate significant differences (*p* < 0.05) between values in one column. Different superscript lowercase letters indicate significant differences (*p* < 0.05) between the same botanical source.

**Table 5:** RVA results for native and acid-treated cassava, quinoa, and faba bean starches at 6 h and 24 h processing time.

Samples		Peak Viscosity (cP)	Breakdown Viscosity (cP)	Setback Viscosity (cP)	Final Viscosity (cP)
Cassava	Native	4475.0 ± 84.9 ^Aa^	2740.0 ± 21.2 ^Aa^	3021.0 ± 93.3 ^Ba^	4756.0 ± 29.7 ^Ba^
	AT6	100.0 ± 4.2 ^Db^	57.0 ± 1.4 ^CDb^	1245.0 ± 21.2 ^Cb^	1288.0 ± 18.4 ^Db^
	AT24	19 ± 1.4 ^Db^	6.5 ± 2.1 ^EFb^	226.5 ± 4.9 ^Ec^	230.0 ± 5.7 ^Ec^
Quinoa	Native	3242.0 ± 60.8 ^Ba^	86.0 ± 15.6 ^Ca^	790.0 ± 7.1 ^Da^	3946.5 ± 37.5 ^Ca^
	AT6	95.5 ± 2.1 ^Db^	42.0 ± 5.7 ^DEb^	92.5.0 ± 3.5 ^Eb^	148.0 ± 28 ^Eb^
	AT24	14.5 ± 0.7 ^Db^	1.0 ± 0.0 ^Fc^	60.0 ± 4.2 ^Ec^	73.5 ± 4.9 ^Ec^
Faba bean	Native	2956.0 ± 36.8 ^Ca^	806.0 ± 8.5 ^Ba^	6194.5 ± 176.1 ^Aa^	8344.5 ± 204.4 ^Aa^
	AT6	117.5 ± 4.9 ^Db^	18.0 ± 4.2 ^EFb^	1070.5 ± 98.3 ^CDb^	1280.0 ± 56.6 ^Db^
	AT24	34.5 ± 3.5 ^Db^	8.5 ± 4.9 ^EFb^	43.0 ± 2.8 ^Ec^	69.0 ± 1.4 ^Ec^

Numerical values are presented in mean ± standard deviation. Different superscript uppercase letters indicate significant differences (*p* < 0.05) between values in one column. Different superscript lowercase letters indicate significant differences (*p* < 0.05) between the same botanical source.

**Table 6:** TPA results for native and acid-treated cassava, quinoa, and faba bean starches at 6 h and 24 h processing time.

Samples		Firmness (g)	Cohesiveness
Cassava	Native	Gel was loose and irregular	GEL was loose and irregular
	6 h	7333.9 ± 1449.3 ^Aa^	0.40 ± 0.03 ^Aa^
	24 h	7087.0 ± 2047.8 ^Aa^	0.40 ± 0.04 ^Aa^
Quinoa	Native	Gel was irregular	Gel was irregular
	6 h	Gel was irregular	Gel was irregular
	24 h	Gel was irregular	Gel was irregular
Faba bean	Native	1536.7 ± 88.2 ^Ba^	0.89 ± 0.04 ^Aa^
	6 h	1255.6 ± 175.4 ^Ba^	0.74 ± 0.12 ^Aa^
	24 h	316.8 ± 53.9 ^Cb^	0.56 ± 0.06 ^Ab^

Numerical values are presented in mean ± standard deviation. Different superscript uppercase letters indicate significant differences (*p* < 0.05) between values in one column. Different superscript lowercase letters indicate significant differences (*p* < 0.05) between the same botanical source.

## Data Availability

The original contributions presented in the study are included in the article, further inquiries can be directed to the corresponding author.

## Abbreviations

C-N	Native cassava starch
C-AT6	Acid-treated cassava starch treated for 6 h
C-AT24	Acid-treated cassava starch treated for 24 h
Q-N	Native quinoa starch
Q-AT6	Acid-treated quinoa starch treated for 6 h
Q-AT24	Acid-treated quinoa starch treated for 24 h
F-N	Native faba bean starch
F-AT6	Acid-treated faba bean starch treated for 6 h
F-AT24	Acid-treated faba bean starch treated for 24 h
SEM	Scanning electron microscopy
DSC	Differential scanning calorimetry
RVA	Rapid visco analyser
TPA	Texture profile analysis
To	Onset temperature from DSC analysis
Tp	Peak temperature from DSC analysis
Tc	Conclusion temperature from DSC analysis
